# Supplementary material for: Requirements for data integration platforms in biomedical research networks: a reference model
Source: PeerJ. 2015 Feb 5;3:e755. doi: 10.7717/peerj.755 (PMC4327254; doi:10.7717/peerj.755)
Supplement: Table S1 [file peerj-03-755-s001.pdf]

Reference goals

Feature	Explanation
Number	RG1
Name	Conduct research project
Description	The top-level goal of a research network is to conduct its intended research project. Often, this goal is provided by a funding agency.
Weighting	high

Feature	Explanation
Number	RG2
Name	Answer research questions
Description	A research network has research questions that are either explicitly or implicitly stated. They guide the direction of the network's research. One goal of the research network is to answer these questions.
Weighting	high

Feature	Explanation
Number	RG3
Name	Create, store, and retrieve data
Description	During the course of a research network's work, data are created. Analysis of these data contributes to the answers of research questions. These data are stored and provided for retrieval by project members.
Weighting	high

Feature	Explanation
Number	RG4
Name	Analyze data
Description	Raw data contributed to a research network are per se without meaning. They have to be analyzed and transformed into knowledge first to contribute to answering research questions and thus supporting the goals of the network.
Weighting	high

Feature	Explanation
Number	RG5
Name	Control data access and usage
Description	Data of the research network require adequate protection. On the one hand, personal data like patient data are affected. On the other hand, unpublished data can be considered as the intellectual property of their creator.
Weighting	medium

Reference requirements

Feature	Explanation
**Number**	RR1
**Name**	Create data
**Description**	The IT system has to provide the capability to create new data or incorporate newly created data. This is necessary as a first step when creating new knowledge.
**Weighting**	high

Feature	Explanation
**Number**	RR2
**Name**	Retrieve external data
**Description**	External data sources must be made available to the research network. Such data sources are for example databases available over the Internet.
**Weighting**	medium

Feature	Explanation
**Number**	RR3
**Name**	Represent data
**Description**	Data of internal or external sources available through the IT system have to be transferred into a suitable representation. This representation has to be precisely defined and locating data must be possible.
**Weighting**	high

Feature	Explanation
**Number**	RR4
**Name**	Define syntax
**Description**	The data format must be syntactically defined in a comprehensive way. This is necessary for correct retrieval and analysis of the network's data sources.
**Weighting**	high

Feature	Explanation
**Number**	RR5
**Name**	Define data model
**Description**	Both a logical and a physical data model are required for efficient internal administration of the research data.
**Weighting**	high

Feature	Explanation
Number	RR6
Name	Identify data
Description	Data sources must be identified unambiguously throughout the research network. Identity and location of the data source must be announced for the network, for example by means of a metadata directory.
Weighting	low

Feature	Explanation
Number	RR7
Name	Define semantics
Description	Semantics of the data provided by the network's data sources must be precisely defined. As a consequence, data integration on a semantic layer is possible.
Weighting	medium

Feature	Explanation
Number	RR8
Name	Administrate intellectual property
Description	Access to data of the research network must be regulated in terms of intellectual property. This is an important prerequisite for the broad acceptance of such data integration platform.
Weighting	medium

Feature	Explanation
Number	RR9
Name	Protect data
Description	Data must be protected against unauthorized access. This is especially important, if personal data like patient data are affected. The conformance with corresponding legal aspects is also covered by this requirement.
Weighting	high

Feature	Explanation
Number	RR10
Name	Show results
Description	For best possible support of researchers data and results of analysis have to be prepared and visualized adequately. The choice of the visualization technique depends on the users' requirements as well as the nature of the data.
Weighting	high

Feature	Explanation
**Number**	RR11
**Name**	Integrate data
**Description**	For analyses of data contributed by more than one project of the research network it is necessary to integrate the data first. For a specific analysis, the adequately represented data are merged.
**Weighting**	medium

Feature	Explanation
**Number**	RR12
**Name**	Define analytical methods
**Description**	For working on research questions, data have to be analyzed with a method that suits the intended purpose. Methods must be chosen from already available approaches or have to be developed for the project.
**Weighting**	medium

Feature	Explanation
**Number**	RR13
**Name**	Define analytical process
**Description**	The process of data analysis is comprised of one or more data sources and one or more analytical methods. These components can be chained and connected to fulfill research tasks.
**Weighting**	high

Feature	Explanation
**Number**	RR14
**Name**	Static Workflow
**Description**	The system provides predefined workflows for analysis. Users are guided since they can only change parameters within the workflow. The system has to ensure, that only sensible parameter combinations are possible.
**Weighting**	medium

Feature	Explanation
**Number**	RR15
**Name**	Dynamic Workflow
**Description**	Users of the IT system have to be able to define analytical pipelines on their own. They are not limited by the system in terms of defining the workflow.
**Weighting**	medium
