# Supplementary material for: Requirements for data integration platforms in biomedical research networks: a reference model
Source: PeerJ. 2015 Feb 5;3:e755. doi: 10.7717/peerj.755 (PMC4327254; doi:10.7717/peerj.755)
Supplement: Table S2 [file peerj-03-755-s002.pdf]

Goals of the research network

Feature	Explanation
**Number**	G1
**Name**	Conduct research project
**Description**	The main goal of the SFB/TRR77 project on liver cancer is to get a deeper understanding of the molecular basis of the corresponding pathogenesis. This includes aspects like<br>• The beginning of the chronic liver disease<br>• Progression<br>• Final cancer stadium with metastases<br>Further, the research network aims to identify new preventive, diagnostic and therapeutic approaches.
**Weighting**	high

Feature	Explanation
**Number**	G2
**Name**	Answer research questions
**Description**	The research network covers, among others, the following research questions:<br>• Which generic or specific mechanisms of chronic liver diseases and inflammatory processes predispose or initiate liver cancer?<br>• Which molecular key events that promote or maintain can be used as tumor markers?<br>• Which are promising targets for future therapies?
**Weighting**	high

Feature	Explanation
**Number**	G3
**Name**	Create, store, and retrieve data
**Description**	The network has the goal to provide data associated with liver cancer. These data include microarray data like aCGH, methylation, or gene expression. Further, imaging data like tissue microarrays and clinical data are considered.
**Weighting**	high

Feature	Explanation
**Number**	G4
**Name**	Analyze data
**Description**	Data of participating projects have to be made available for cross-project analyses.
**Weighting**	high

Feature	Explanation
**Number**	G5
**Name**	Control data access and usage
**Description**	Data of SFB/TRR77 have to be protected. Depending on the type of data, only specific collaborators of the research network, all members of the network, or the general public can access data.
**Weighting**	high

Requirements of the research network

Feature	Explanation
**Number**	R1
**Name**	Create data
**Description**	Within the research network, mircroarray data on liver cancer specimen are produced. These data are not created within the IT platform itself, but distributed. Individual projects generate data for their own research. If required, these data are shared within the research network. This leads to the additional requirement of preparing the data for distribution.
**Weighting**	high

Feature	Explanation
**Number**	R2
**Name**	Retrieve external data
**Description**	Genomic data created within the network have to be annotated in terms of functionality and roles in regulatory pathways. This has to be done by correlating data bases available over the Internet like gene ontology or KEGG.
**Weighting**	medium

Feature	Explanation
**Number**	R3
**Name**	Represent data
**Description**	Data of the research network have to be easily accessible and locatable to enable their correlation. Thus, a central pint of data access is required.
**Weighting**	high

Feature	Explanation
**Number**	R4
**Name**	Define syntax
**Description**	Syntax of the data available via the IT platform of the network has to be defined and documented in a suitable way. For data and analytical services, XML documents according to the WSDL specification are required.
**Weighting**	high

Feature	Explanation
**Number**	R5
**Name**	Define data model
**Description**	For all data accessible via the central IT platform data models have to be specified and documented. This is a prerequisite for data harmonization. For certain aspects documentation according to the UML-XMI specification is required.
**Weighting**	medium

Feature	Explanation
**Number**	R6
**Name**	Identify data
**Description**	All data sources need to be annotated with a unique URI to make them identifiable and accessible throughout the research network.
**Weighting**	low

Feature	Explanation
**Number**	R7
**Name**	Define semantics
**Description**	Semantics of the data provided for the SFB/TRR77 network have to be defined precisely by the data contributors. This is a prerequisite for cross-project data analysis.
**Weighting**	medium

Feature	Explanation
**Number**	R8
**Name**	Administrate intellectual property
**Description**	Projects of the research network require maintaining control over data they share via the IT platform. Further, suitable mechanisms for ensuring credit for data contributors have to be established.
**Weighting**	high

Feature	Explanation
**Number**	R9
**Name**	Protect data
**Description**	Data must be protected against unauthorized access. Since no identifying personal data are stored on the IT platform, this requirements helps mostly to enforce R8.
**Weighting**	medium

Feature	Explanation
**Number**	R10
**Name**	Show results
**Description**	Results of the network's data analyses have to be visualized in a central place. Different kinds of presentation, like tables or diagrams, have to be available.
**Weighting**	high

Feature	Explanation
**Number**	R11
**Name**	Integrate data
**Description**	Data of the research network have to be analyzed across project borders. Thus, they have to be integrated and made available for the whole network.
**Weighting**	medium

Feature	Explanation
**Number**	R12
**Name**	Define analytical methods
**Description**	For the correct analysis of the research network's data, specific biostatistical methods have to be selected and established. Flexibility is required to support a wide range of data types and alternative approaches.
**Weighting**	medium

Feature	Explanation
**Number**	R13
**Name**	Define analytical process
**Description**	The process of data analysis is comprised of one or more data sources and one or more analytical methods. These components have to be chained and connected to fulfill research tasks of the network.
**Weighting**	high

Feature	Explanation
**Number**	R14
**Name**	Static Workflow
**Description**	The IT platform has to provide predefined workflows for data analysis. Users have to be able to parameterize the workflow to run a specific analysis. Parameters could include gene symbols or statistical methods that can be chosen from a list.
**Weighting**	medium

Feature	Explanation
**Number**	R15
**Name**	Dynamic Workflow
**Description**	The IT platform has to provide tools for the users to design complete analytical workflows themselves. This includes selecting the available data and analytical services and chaining them into arbitrary pipelines.
**Weighting**	low
